# Comfortable Flower Electrodes for Dry EEG in Epilepsy and Clinical Neurophysiology Diagnostics

**DOI:** 10.3390/s26072146

**Published:** 2026-03-31

**Authors:** Dimitrios Dimitrakopoulos, Justus Marquetand, Joji Kuramatsu, Patrique Fiedler, Johannes Lang

**Affiliations:** 1 Department of Neural Dynamics and Magnetoencephalography, Hertie-Institute for Clinical Brain Research, University of Tuebingen, 72076 Tuebingen, Germanyjustus.marquetand@cerebri-health.com (J.M.); 2 Institute for Modelling and Simulation of Biomechanical Systems, University of Stuttgart, 70569 Stuttgart, Germany; 3 Cerebri GmbH, Neckargasse 7, 72070 Tuebingen, Germany; 4 Department of Neurology, RoMed Clinic Rosenheim, Ellmaierstraße 23, 83022 Rosenheim, Germany; joji.kuramatsu@ro-med.de; 5 Department of Neurology, University of Erlangen-Nuremberg, Schwabachanlage 6, 91054 Erlangen, Germany; 6 Institute of Biomedical Engineering and Informatics, Technische Universität Ilmenau, Gustav-Kirchhoff-Str. 2, 98693 Ilmenau, Germany; patrique.fiedler@tu-ilmenau.de

**Keywords:** dry electrode, electrophysiology, electroencephalography, gel-free

## Abstract

**Highlights:**

**What are the main findings?**

- Novel dry electrodes, featuring a flower design (“flower electrodes”), offer a sufficient signal-to-noise ratio (SNR) for clinical neurophysiological diagnostics.
- The electrodes were tested in a clinical setting with three patients with epilepsy and delirium, providing preliminary evidence of feasibility.

**What are the implications of the main findings?**

- This new electrode design provides a more comfortable, gel-free, and resource-efficient alternative to traditional EEG electrodes.
- It has the potential to improve point-of-care EEG diagnostics and patient compliance during long-term monitoring and routine clinical EEG recordings.

**Abstract:**

Dry electroencephalography (EEG) electrodes enable rapid, gel-free setups, which are crucial for point-of-care diagnostics, but often face challenges with comfort and signal quality—especially in a clinical context. Novel “flower” dry electrodes are a special type of reusable scalp electrodes for dry EEG, featuring a distinct flower-like shape with angled pins in three intertwined layers. While the new electrode design has been validated in an in vivo study on healthy volunteers, we tested its clinical applicability in a proof-of-concept study involving three patients diagnosed with epilepsy and delirium. The recordings were of high diagnostic quality, enabling the reliable identification of pathological patterns, such as generalized spike–wave complexes and intermittent delta activity, with a signal-to-noise ratio comparable to prior reports for sponge-based EEG systems (limited case series). The signal-to-noise ratio (SNR) proved to be sufficiently high for clinical diagnostic purposes, resulting in visually clear and interpretable EEG data that enabled effective assessment of patients’ neurophysiological signals. Consequently, our findings demonstrate that the comfortable flower-electrode design is a viable and effective tool for epilepsy diagnostics, extended recording, and clinical neurophysiology. It represents a significant step towards patient-centered and gel-free EEG technology, specifically in point-of-care and emergency applications, without compromising the diagnostic quality of the recordings.

## 1. Introduction

Electroencephalography (EEG) remains a cornerstone of clinical neurophysiology, being critical for diagnosing and managing neurological disorders such as epilepsy [1], non-convulsive status epilepticus (NCSE) [2], or encephalopathies [3]. Particularly, the identification of specific discharges and patterns such as spike–wave complexes (SWCs) or slow-wave activity is crucial for informed clinical decision-making. However, the widespread use of EEG, particularly in point-of-care and emergency situations [4,5], is often limited by the drawbacks of traditional electrode technology, which necessitates (1) abrasive skin preparation [6], (2) the use of conductive gel, and (3) a proper understanding of electrode placement and control. Whereas knowledge of electrode placement can be overcome by the use of electrode nets with predefined sensor positions, the process of patient skin preparation and applying gel is time-consuming, causes patient discomfort, and is susceptible to signal degradation over long durations as the gel dries [7]. What is needed are EEG electrode systems that do not require trained personnel nor long preparation time [8]. This is specifically crucial for resource-limited settings, emergency situations [9], during off-hours, or at patients’ homes. Consequently, there is a high unmet medical need for an electrode technology that can be applied quickly, easily, and by inexperienced personnel without compromising signal quality.

The development of dry electrodes has emerged as a promising solution to enable rapid, gel-free setups [10,11,12,13]. However, many existing designs present a new set of challenges. They must often compromise between signal quality—achieving a stable, low-impedance contact—and patient comfort, especially during extended wear or with sensitive populations [14,15]. This trade-off is particularly critical in a clinical context where patient compliance and high-fidelity data are needed. Alternative quick-apply systems have been explored. Sponge-based EEG (sp-EEG) systems, which utilize saline-soaked sponges integrated with silver/silver-chloride (Ag/AgCl) disk electrodes and into a cap, have been shown to be rapidly applicable and provide signal quality equivalent to routine EEG (r-EEG). However, the necessity of a pre-soaking step and a conductive liquid remains, thus exhibiting time-dependent impedance and signal quality levels. Other systems, such as those using dry electrodes with pin-cushion designs to overcome hair barriers, have demonstrated potential but can be uncomfortable, depending on the intensity of the pressing force and the hardness of the material [16,17,18,19].

**Figure 1:**
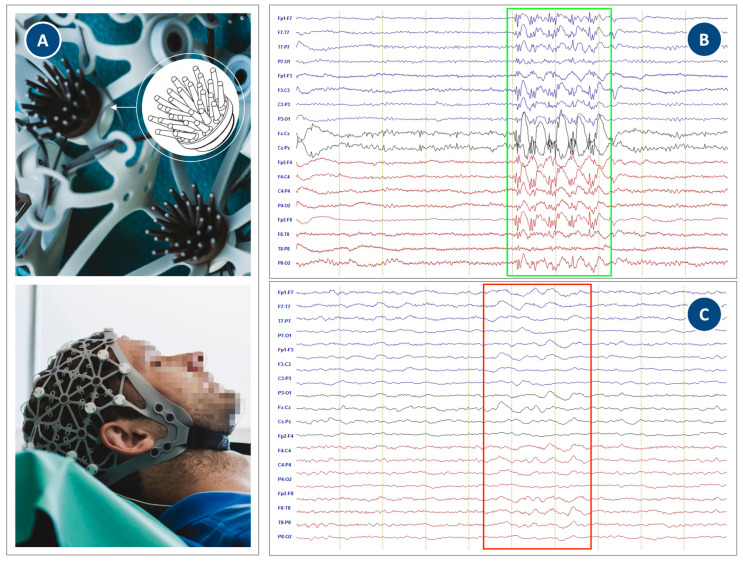
Representative EEG traces from the flower electrode system. (**A**) Illustration of the flower EEG components from top to bottom: electrode design; electrodes integrated into the silicone net cap structure; cap worn by a volunteer (for illustration; model). (**B**) Patient #1: Generalized spike–wave complexes (SWCs) are clearly visible (green rectangle). (**C**) Patient #2: Intermittent rhythmic delta activity in the fronto-temporal regions (red rectangle).

To bridge the gap between ease of use, comfort, and diagnostic fidelity, we present a proof-of-concept clinical evaluation of the novel “flower” dry electrode. This design, characterized by multiple flexible, angled pins in a flower petal-like arrangement (see Figure 1A), aims to increase the contact area and enhance wearing comfort while maintaining the ease of use inherent to dry systems. In previous studies the detailed development and a lab-based validation on healthy volunteers were successfully performed. For a complete technical description and characterization, please refer to Warsito et al. [18]. Based on the previous findings, in this communication we aim to evaluate the performance of the same electrodes in a new field of application. We report on the deployment of a custom EEG-net equipped with these flower electrodes on three patients undergoing diagnostic monitoring for epilepsy and delirium. Our objective was to validate the system’s clinical applicability for the first time by evaluating its ability to accurately record neurophysiological signals required for standard clinical practice.

## 2. Materials and Methods

### 2.1. Study Population

Three patients were prospectively recruited from the worklist for EEG diagnostics at the Department of Neurology at the RoMed Clinic Rosenheim. The cohort included individuals undergoing diagnostic workup for suspected epilepsy or delirium with transient altered mental status. Demographic and clinical details are summarized in Table 1. The study was conducted in accordance with the principles of the Declaration of Helsinki. Data was recorded anonymized, i.e., their EEG, and clinical and demographic data. The Ethics Committee of the University of Tübingen reviewed the study protocol and granted a waiver of ethical approval (reference number 2025-0822-A), as data were recorded anonymously and procedures were performed under standard clinical care.

### 2.2. Flower Electrode System and Measurement Procedure

A custom 24-channel EEG cap integrating the novel flower dry electrodes was used for the study. The flower electrode design features multiple, flexible silver-coated pins arranged in a circular, flower-like pattern (see Figure 1A). The design is implemented using a flexible polyurethane substrate of shore hardness A65 for frontal electrodes (Fp1 and Fp2) and A98 for all other electrodes, coated with silver/silver chloride. For a detailed description of the electrode design, optimization and characterization under lab conditions, please refer to Warsito et al. [18]. The previous studies demonstrated that the electrode pins gently navigate through hair while maintaining stable skin contact with minimal pressure, thereby improving comfort. The cap was positioned on the head according to the 10–20 system and secured with a chin strap. Electrode contact was verified via the system’s impedance check, with values considered acceptable per the manufacturer’s instructions (typically <250 kΩ for dry electrode systems). Reference (REF) and Ground (GND) electrodes were placed on the mastoids using multi-purpose monitoring sticky electrode pads. Resting EEG was recorded for approximately 20 min per patient with eyes open and closed according to the recommendations of the German Society for Clinical Neurophysiology and Functional Imaging (DGKN, guideline #4) for routine EEG with a sampling rate of 500 Hz using the eego^TM^ 24 amplifier (ANT Neuro, Hengelo, The Netherlands; input impedance > 1 GΩ) on a nëo^TM^ all-in-one panel PC and software (version 01.05.01.62282, eemagine Medical Imaging Solutions, Berlin, Germany). During recording provocative maneuvers such as photic stimulation and hyperventilation were performed where clinically feasible. Bipolar (longitudinal double banana) and reference (Cz) montages were used during recording and reviewing. Since the nëo software (version 01.05.01.62282) features continuous live impedance per channel, no final impedances were obtained.

### 2.3. Signal Quality and Visual Analysis

Recorded signals were band-pass filtered (0.3–70 Hz), and a 50 Hz notch filter was applied. EEGs were visually inspected by two experienced, board-certified clinical neurophysiologists following a blinded protocol. They were asked to identify physiological (e.g., posterior dominant rhythm, reactivity) and pathological graphoelements (e.g., spikes, sharp waves, slowing). Furthermore, raters were asked to comment on the overall signal quality and interpretability for clinical diagnosis.

The signal-to-noise ratio (SNR) was computed for each spike–wave complex (SWC) in one fully cooperative patient (Patient #1) using an FFT-based approach analogous to [20]. For each SWC k, FFT-based power spectral densities (PSDs) were calculated in BESA from a 1 s SWC window and a matched 1 s baseline window (per channel). Band power was defined as the area under the PSD within the respective frequency limits, yielding δ, θ, α, β and γ. The SNR was then derived as the channel-summed increase in δ-band power from baseline to SWC, normalized by the channel-summed non-δ band power (θ + α + β + γ) across the SWC and baseline windows, resulting in an SNR_k_ time series across events, as shown in the formula below.

$${\mathrm{SNR}}_{k}=\frac{\sum_{c=1}^{N_{\mathrm{ch}}}\left(\delta_{\mathrm{SWC}}^{\left(c,k\right)}-\delta_{0}^{\left(c,k\right)}\right)}{\sum_{c=1}^{N_{\mathrm{ch}}}\left[\left(\theta_{\mathrm{SWC}}^{\left(c,k\right)}+\alpha_{\mathrm{SWC}}^{\left(c,k\right)}+\beta_{\mathrm{SWC}}^{\left(c,k\right)}+\gamma_{\mathrm{SWC}}^{\left(c,k\right)}\right)+\left(\theta_{0}^{\left(c,k\right)}+\alpha_{0}^{\left(c,k\right)}+\beta_{0}^{\left(c,k\right)}+\gamma_{0}^{\left(c,k\right)}\right)\right]}$$

To assess whether the SNR changed systematically over consecutive SWCs, we fitted an ordinary least-squares linear model SNR_k_ = β_0_ + β_1_k. The primary hypothesis of interest was a decrease over time (β_1_ < 0); in addition, we performed a non-inferiority test against a prespecified margin of Δ = −0.03 in the SNR per event (i.e., testing β_1_ > −0.03).

## 3. Results

The Flower electrode cap was successfully applied to all three patients. Application time, measured from cap handling to signal acquisition with acceptable impedances, was rapid, averaging at only two minutes per patient compared to a mean application time of 12 min for bridge electrodes in comparable patients and a clinical situation within the same EEG lab. No difference in signal due to dirty hair could be observed. However, given the small sample size, we could not comprehensively assess the impact of hair and skin conditions (e.g., oily, dirty, sweaty, dry), ambient temperature (cold or warm), or the use of hair products (e.g., hairspray or gel). The system was well tolerated by all patients, including the elderly, confused individual (Patient 2), who made only little attempt to remove the cap.

### 3.1. Signal Quality and Clinical Interpretation

Visual inspection of the EEG recordings by blinded and board-certified experts (JL, JM) confirmed high signal quality; i.e., critically, pathological graphoelements were reliably detected: In Patient #1, generalized SWCs were clearly visible (Figure 1B). Patient #2 showed intermittent fronto-temporal delta slowing, consistent with an encephalopathic state (Figure 1C). For Patient #3, who showed an unremarkable EEG, the averaged resting-state power spectral density (PSD) for frequencies of 1–30 Hz computed from artifact-free 1 s windows for each channel showed the expected 1/f decay with an alpha peak and no abnormal low-frequency dominance or high frequency elevation (Supplementary Figure S1). The blinded raters confirmed that the recordings were of sufficient quality for definitive clinical interpretation. No systematic artifacts or quality deficits could be found that would differentiate these recordings from those obtained with conventional wet or sponge-based EEG systems.

### 3.2. Signal-to-Noise Ratio

**Figure 2:**
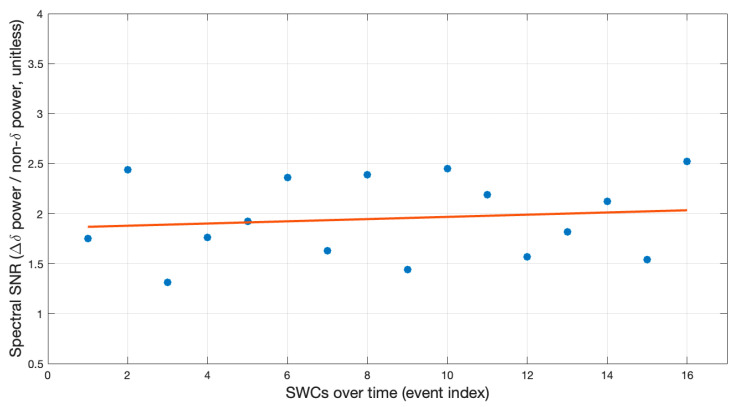
Spectral signal-to-noise ratio (SNR), based on spike–wave complexes (SWCs) across a 20 min EEG recording. No evidence of decreasing SNR over events (slope = 0.011 per event, 95% CI −0.037 to 0.059), and non-inferiority to a decline of −0.03 per event (one-sided *p* = 0.044; lower bound −0.0285).

The quantitative analysis in Patient #1 (with diagnosed SWC) yielded a mean SNR of 1.95 (SD 0.4) for the flower electrode system, which is of the same order of magnitude as the FFT-derived SNR values reported for sponge-based EEG systems [20]. The SNR did not show a decreasing trend across 16 events (slope (β_1_) = 0.011 SNR/event, 95% CI −0.037 to 0.059; one-sided *p* = 0.685) (Figure 2). Using a non-inferiority margin of −0.03 in SNR per event, a decline was unlikely to exceed this threshold (*p* = 0.044; one-sided 95% lower bound −0.0285).

## 4. Discussion

Our proof-of-concept study demonstrates that the novel flower dry electrode system is feasible for clinical use and provides signal quality sufficient for identifying key pathological graphoelements in patients with epilepsy and encephalopathy. The main findings are as follows: First, the flower electrode design successfully bridged the gap between patient comfort and signal quality. The comfortable, gel-free application was well tolerated, even by a patient with confusion (Patient #2), potentially improving compliance and diagnostic yield in challenging cohorts. Second, the application was rapid, underlining its utility in point-of-care and emergency settings where time and personnel are limited. Third, and most importantly, the diagnostic quality was uncompromised. Expert board-certified reviewers identified all relevant physiological and pathological patterns, confirming the system’s clinical validity, without deterioration of SNR for pathological graphoelements over time. These findings align with and extend previous research on alternative EEG systems. While sponge-EEG allows for rapid application and high signal quality, it still relies on a liquid conductor that dries over time and may cause discomfort or distraction for patients. Dry electrode systems have faced challenges due to higher impedances and low wearing comfort due to material rigidity, especially in the sensitive areas of the scalp. The flower electrode’s design appears to mitigate these issues by combining a comfortable, petal-shaped, multipin contact system with flexible material characteristics and active shielding technology, achieving a favorable SNR despite potentially higher nominal impedances.

A notable advantage was the stability of the cap system during patient movement, a finding comparable to observations reported for sponge-EEG [20]. The fixed electrode positions within the cap also standardize placement and reduce inter-operator variability, which is a significant benefit for inexperienced users. A limitation of the cap system, shared with other preconfigured caps [21], is the inability to adapt to pronounced head deformities (e.g., post-hemicraniectomy [22]), where traditional gel-based EEG with individually placed electrodes offers advantageous flexibility.

Future work should test these results in larger and more diverse cohorts and under a variety of recording durations, including systematic quantification of (i) diagnostic sensitivity taking into account other specific graphoelements and patterns (e.g., IEDs, periodic discharges, and seizures), (ii) motion robustness and artifact susceptibility, and (iii) performance over time (e.g., signal stability, comfort, and potential drift) compared directly against gel-based and sponge-based systems using harmonized metrics [12]. Furthermore, future studies should directly compare electrode placement accuracy (e.g., inter-operator variability) and recording quality across systems when applied by non-expert personnel in realistic ICU and emergency department workflows [23].

## 5. Conclusions

The flower dry electrode system is a promising, comfortable tool for clinical neurophysiology. It enables rapid, gel-free EEG acquisition without compromising diagnostic quality, thereby facilitating point-of-care diagnostics and long-term monitoring. These promising findings warrant further validation in larger, direct comparison studies.

## Figures and Tables

**Table 1:** Demographic, clinical and EEG details of patients. * Time until each of the 18 electrodes reached a sufficient impedance of 100 kOhm.

Patient No	Gender (f/m)	Age (Years)	Clinical Indication	EEG Diagnosis	Apply Time * (s)
#1	f	40	Monitoring of disease activity in generalized epilepsy	Generalized spike–wave complexes (SWCs)	67
#2	m	84	Exclusion of a non-convulsive status epilepticus	Intermittent rhythmic delta activity in the fronto-temporal region	122
#3	m	86	Differential diagnosis focal vs. generalized epilepsy	Unremarkable	89

## Data Availability

Data will be made available upon reasonable request.

## Supplementary Materials

The following supporting information can be downloaded at: https://www.mdpi.com/article/10.3390/s26072146/s1, Figure S1: Resting-state PSD in Patient #3 (unremarkable EEG). PSDs for the displayed clinically relevant frequencies (1–40 Hz) were computed for each channel in 90 artifact-free non overlapping 1 s windows (using Welch’s method, 1 s Hamming window) and finally averaged for the total PSD estimate per channel.

## Abbreviations

The following abbreviations are used in this manuscript:

EEG	Electroencephalogram
SNR	Signal-to-noise ratio
SWC	Spike–wave complex
