# Supplementary material for: Comfortable Flower Electrodes for Dry EEG in Epilepsy and Clinical Neurophysiology Diagnostics
Source: Sensors (Basel). 2026 Mar 31;26(7):2146. doi: 10.3390/s26072146 (PMC13074899; doi:10.3390/s26072146)
Supplement: Supplementary file 1 [file sensors-26-02146-s001.zip › sensors-4165260-supplementary.pdf]

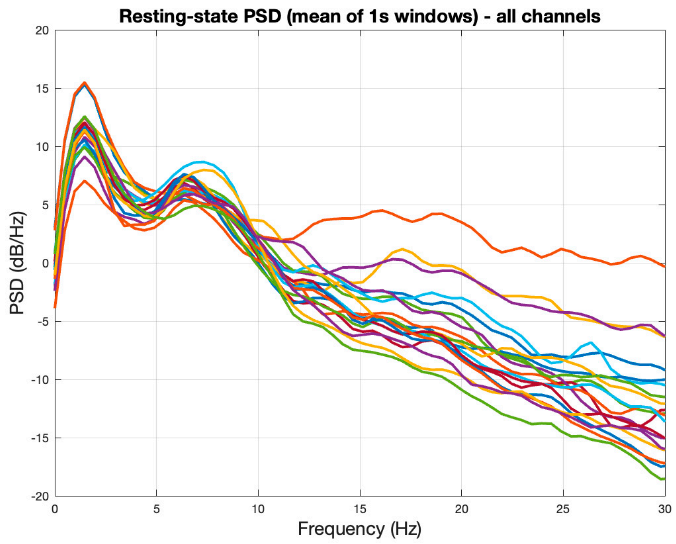

Supplementary Figure S1. Resting-state PSD in Patient #3 (unremarkable EEG). PSDs for the displayed clinically relevant frequencies (1-40 Hz) were computed for each channel in 90 artifact-free non overlapping 1-second windows (using Welch's method, 1 s Hamming window) and finally averaged for the total PSD estimate per channel.
